# Adjuvant medical therapy in cervical dystonia after deep brain stimulation: A retrospective analysis

**DOI:** 10.3389/fneur.2022.927573

**Published:** 2022-08-05

**Authors:** Alfonso E. Martinez-Nunez, Christos Sidiropoulos, Julia Wall, Jason Schwalb, Ellen Air, Peter LeWitt, Bisena Bulica, Patricia Kaminski, Neepa Patel

**Affiliations:** ^1^Department of Neurology, Henry Ford Hospital, Detroit, MI, United States; ^2^Department of Neurology and Ophthalmology, Michigan State University, East Lansing, MI, United States; ^3^Department of Neurosurgery, Henry Ford Hospital, Detroit, MI, United States; ^4^Department of Neurology, Wayne State University School of Medicine, Detroit, MI, United States; ^5^Parkinson's Disease and Movement Disorders Program, Department of Neurological Sciences, Rush University Medical Center, Chicago, IL, United States

**Keywords:** cervical dystonia, deep brain stimulation, medical therapy, botulinum toxin, long-term follow up

## Abstract

**Background:**

There is limited information on optimization of symptomatic management of cervical dystonia (CD) after implantation of pallidal deep brain stimulation (DBS).

**Objectives:**

To describe the long-term, “real-world” management of CD patients after DBS implantation and the role of reintroduction of pharmacologic and botulinum toxin (BoNT) therapy.

**Methods:**

A retrospective analysis of patients with focal cervical or segmental craniocervical dystonia implanted with DBS was conducted.

**Results:**

Nine patients were identified with a mean follow-up of 41.7 ± 15.7 months. All patients continued adjuvant oral medication(s) to optimize symptom control post-operatively. Three stopped BoNT and four reduced BoNT dose by an average of 22%. All patients remained on at least one medication used to treat dystonia post-operatively.

**Conclusion:**

Optimal symptom control was achieved with DBS combined with either BoNT and/or medication. We suggest utilization of adjuvant therapies such as BoNT and/or medications if DBS monotherapy does not achieve optimal symptom control.

## Introduction

Dystonia is defined as “a movement disorder characterized by sustained or intermittent muscle contractions causing abnormal, often repetitive, movements, postures, or both” (1). Cervical dystonia (CD) is the most common form of adult focal dystonia characterized by sustained or intermittent muscle contractions of neck muscles that result in involuntary intermittent or sustained posturing of the head.

CD can be associated with significant disability, pain and reduced quality of life. Though botulinum toxin (BoNT) is the standard of care for treatment of CD, up to one-third of CD patients have suboptimal therapeutic response (2). Development of neutralizing antibodies, short duration of benefit between BoNT injections, side effects and delays between injections often contribute to suboptimal treatment response (3). Medications such as anticholinergics, muscle relaxants and benzodiazepines, as well as physical therapy (4), are often used as adjunctive therapies with variable effectiveness (5). Deep brain stimulation (DBS) has become a safe and effective therapy for management of medically refractory CD (6). There are only a few studies describing the long-term effects of DBS on CD beyond 5 years, with reports of an average of at least 25–50% improvement of motor symptoms of CD (7–9). Though this is a clinically meaningful improvement, many patients continue to struggle with pain and spasms in the neck which are not fully controlled with DBS monotherapy. There is limited information related to strategies for optimizing symptomatic benefit in CD beyond DBS monotherapy for those patients experiencing less symptomatic benefit.

We aim to describe the long-term management of CD patients who underwent DBS at the Henry Ford Hospital Movement Disorders Clinic.

## Methods

This is a retrospective chart review of medication-refractory CD patients treated with DBS. Patients with isolated CD that were followed up for at least 1 year postoperatively were identified from the Henry Ford Hospital Movement Disorders Clinic database. Patients with hemidystonia or generalized dystonia were excluded from this analysis. All of our patients underwent asleep surgery with intraoperative magnetic resonance imaging (iMRI) following the stereotactic coordinates and techniques previously described by Starr (10). A monopolar review was performed on all of our patients during the first office visit follow-up. This study was reviewed and approved by the Henry Ford Health System Internal Review Board (IRB). This study is conducted according to the declaration of Helsinki.

Demographic data, preoperative Toronto Western Spasmodic Torticollis Scale (TWSTRS) scores (obtained within 1 year prior to DBS implantation), duration of therapy, final programming parameters, pharmacologic and BoNT treatments before and after DBS surgery were captured. Comparison of different BoNT formulations were converted to onabotulinum toxin A equivalents as based upon published guidelines (11). Data was collected retrospectively from the last follow-up visit at Henry Ford Health System.

Descriptive statistics (central tendency measures, proportions) were used to describe demographics, predominant CD phenomenology, motor evaluations, stimulation parameters, and use of adjuvant medication. For comparison of means, we initially ran a normality test (Kolmogorov-Smirnov) to decide whether to use a parametric test (student *t*-test) or a non-parametric test (Mann-Whitney *U* test). When comparing two dichotomous variables we calculated an odds ratio (OR) and used the chi-square test to determine independence between categorical polychotomous variables.

## Results

Of the 975 patients with CD in Henry Ford Movement Disorder's clinic database (from January 1, 2014 to April 1, 2020), 11 patients underwent DBS. Two patients were excluded due to their lack of follow-up after the 1^st^ year of surgery. Of the remaining 9 patients, all were implanted with bilateral DBS targeting the globus pallidus internus (GPi). Clinical features of CD are summarized in Table 1. All of our patients were on some form of adjuvant medication and received BoNT injections in cervical muscles pre-operatively, and two of our patients received facial muscles injections for blepharospasm.

**Table 1:** Characteristics of dystonia.

Female	5 of 9 (56%)
Duration of follow up	41.67 ± 15.7 months
Age at onset of disease	46.56 ± 8.2 years
Age at the time of DBS implantation (mean ± standard deviation)	55.8 ± 10.8 years
Duration of CD prior to DBS (mean ± standard deviation)	9.4 ± 8.2 years
Primary direction of dystonic movement	
Laterocollis	5 of 9 (56%)
Torticollis	4 of 9 (44%)
Patients that continue to receive BoNT injections	6 of 9 (67%)

All of our patients required continuation of adjuvant therapies in combination with DBS to attain satisfactory control of their dystonia symptoms post-operatively (Table 2). Six patients continued to receive BoNT injections, 4 remained on anticholinergic medications, 3 on muscle relaxants, and 7 on benzodiazepines. Six of the patients were able to reduce their adjuvant therapies post-operatively, and 7 patients were able to reduce BoNT injections (an average dose reduction of 22%, 75.8 ± 14.2 units of onabotulinum toxin A equivalents), of which 3 patients completely stopped their use. The two patients with blepharospasm were able to cease BoNT injections. There was a significant decrease in the mean number of muscles that were injected per BoNT injection session after DBS implantation (8.4 ± 1.5 vs. 6.1 ± 0.7), *p* = 0.006).

**Table 2:** Individual description of each of our patients including disease phenotype, stimulation parameters and adjuvant medication.

					**Preoperative TWSTRS**					**DBS settings**						
**Patient**	**Age at time of surgery (years)**	**Duration of disease prior to surgery (years)**	**Follow up after surgery (months)**	**Dystonia topographic distribution and predominant direction of dystonic pull**	**Total**	**Motor**	**Disability**	**Pain**	**Psychiatric comorbidities**	**Lead/ polarity**	**Amplitude (V)**	**Pulse width (us)**	**Frequency (Hz)**	**Side-effects from stimulation**	**Adjuvant medication prior to surgery**	**Adjuvant medication at the last time of follow up**
1	59	9	25	Focal / Right laterocollis	-	-	-	-	MDD	Left GPi: C+2–3- Right GPi: C+10–11-	4 4	210 210	130 130	None	Alprazolam 1 mg BID Onabotulinum toxin A (300 U)	Alprazolam 1 mg QID Onabotulinum toxin A (300 U)
2	64	30	56	Focal / Left laterocollis	28	9	16	3	GAD	Left GPi: C+2- Right GPi: C+10-	1.8 2.3	90 90	160 160	Acral dysesthesias	Clonazepam 2 mg TID Onabotulinum toxin A (300 U)	Lorazepam 2 mg QID
3	34	7	51	Segmental (CD and BS) / Right laterocollis	-	-	-	-	ADHD	Left GPi: C+0–1- Right GPi: C+8–9-	4.7 4.6	60 90	130 130	Right arm/ hand cramping and spasms and right foot curling	Trihexyphenidyl 2 mg TID Baclofen 10 mg BID Diazepam 5 mg TID Onabotulinum toxin A (325 U)	Trihexyphenidyl 2 mg TID Baclofen 10 mg qd Onabotulinum toxin A (200 U)
4	59	6	49	Focal / Left laterocollis	39	11	12	16	GAD	Left GPi: C+0–1- Right GPi: C+8–9-	3.6 3.6	180 180	140 140	Blepharospasm	Clonazepam 0.5 mg BID Onabotulinum toxin A (175 U)	Clonazepam 0.5 mg BID Onabotulinum toxin A (255 U)
5	40	11	49	Focal / Left laterocollis and retrocollis	54	24	16	14	None	Left GPi: C+0- Right GPi: C+8-	3 3.3	60 90	130 130	None	Trihexyphenidyl 2 mg TID Rimabotulinum toxin B (17500 U)	Trihexyphenidyl 2 mg TID Baclofen 20 mg TID Diazepam 2 mg TID Onabotulinum toxin A (380 U)
6	64	9	49	Focal / Left torticollis and laterocollis	16	4	4	8	GAD	Left GPi: C+0- Right GPi: C+8-	2.5 2.5	60 60	125 125	None	Trihexyphenidyl 2 MG TID Diazepam 10 mg BID Onabotulinum toxin A (300 U)	Diazepam 10 mg TID
7	53	2	48	Focal / Right torticollis	35	16	11	8	None	Left GPi: C+1- Right GPi: C+9-	3 3	90 90	125 125	None	Trihexyphenidyl 2 mg TID Baclofen 10 mg BID Diazepam 5 mg TID Onabotulinum toxin A (500 U)	Baclofen 10 mg TID Diazepam 5 mg TID Onabotulinum toxin A (500 U)
8	65	3	41	Focal / Right torticollis	31	13	11	7	None	Left GPi: C+1–2- Right GPi: C+9–10-	3 2.6	90 80	130 130	None	Clonazepam 1 mg BID Onabotulinum toxin A (300 U)	Trihexyphenidyl 2 mg TID Onabotulinum toxin A (400 U)
9	65	8	7	Segmental (CD and BS) / Left laterocollis	-	-	-	-	GAD	Left GPi: C+ 3- Right GPi: C+ 2–3-	3.3 3.2	60 60	130 130	None	Trihexyphenidyl 2 mg TID Lorazepam 0.5 mg TID Onabotulinum toxin A (300 U)	Trihexyphenidyl 2 mg TID Lorazepam 0.5 mg TID
**Mean**	55.89 (±10.83)	9.44 (±8.23)	41.67 (±15.7)	-	33.83 (±16.92)	12.83 (±6.79)	11.67 (±4.41)	9.33 (±4.8)	-	-	3.22 (±0.77)	92.78 (±37.07)	133.33 (±10.57)	-	-	-

*TWSTRS, Toronto Western Spasmodic Torticollis Rating Scale; CD, cervical dystonia; BS, blepharospasm; U, units; BID, twice a day; TID, three times a day; QID, four times a day; qd, every day; GPi, globus pallidus pars interna; GAD, generalized anxiety disorder; ADHD, attention-deficit hyperactivity disorder. Standard deviations are in parenthesis*.

A comparative analysis of patients who stopped or decreased BoNT (*n* = 7) vs. patients who kept requiring similar BoNT doses (*n* = 2) was performed. Those patients who were able to decrease or stop BoNT had a longer duration of dystonia symptoms compared to those who remained on similar doses to the pre-operative treatment plan (11.4 ± 8.3 years vs. 2.5 ± 0.7 years, *p* = 0.03).

The average time from implantation to optimization of DBS settings was 11.8 ± 1.7 months. There was no difference in the use of adjuvant medication between patients with or without psychiatric comorbidities (OR = 0.5, CI: 95% −0.3–8.9). Patient 2 and 3 both experienced stimulation induced side effects, localization of the DBS electrodes demonstrated appropriate lead location.

## Discussion

Since the first uses of DBS for CD appeared in the medical literature in 2002, the reported individual responses have been varied (9, 12). Though some patients achieve optimal symptomatic control with DBS monotherapy these findings and our clinical experience suggest that many patients do not. Thus warranting the consideration of adjuvant therapies to optimize symptom control. To our knowledge this study is the first to report the “real-world” long-term management of focal and segmental CD patients with bilateral pallidal DBS in patients previously treated with BoTN therapy. Despite achieving clinically meaningful benefit from DBS therapy, each of our patients continued to require at least one adjuvant therapy to optimize symptom control. DBS facilitated the opportunity to reduce the botulinum toxin dose and/or eliminate some of the muscles previously injected while achieving better symptomatic improvement than pharmacological or BoNT therapy. Of note, most of our patients continue to require benzodiazepines for control of their dystonic symptoms, although comorbid generalized anxiety disorder is another factor that could have favored the ongoing use of this medication class.

Similar to our experience, Yamada et al. reported that eight patients in their cohort also continued adjuvant pharmacological therapies post-operatively, apart from one patient who did not receive medications preoperatively (8). However, in this study the authors did not comment on the use of BoNT pre- or post-operatively. In a prospective study of long-term outcomes with pallidal DBS in all types of dystonia, Krause et al. reported that 42% of their patients were able to reduce or stop their medication. Of the 4 patients in this cohort who received BoNT pre-operatively 3 were able to discontinue therapy and 1 was able to reduce the dose BoNT at last follow-up (13). However, the sub-type of dystonia in relation to the use of medication and BoNT was not reported. Similar to our findings, there was a mean delay of 11.8 months for patients to achieve optimal symptomatic benefit with DBS with similar final stimulation parameters (14). In our study long pulse widths were not found to achieve better symptomatic control in our group of CD patients, as reported by others (15).

A surprising result in this study was the correlation between a longer duration of disease pre-operatively and a larger reduction in BoNT dose used post-operatively. This is counterintuitive to reports of longer disease duration impacting the efficacy of DBS in CD (8). Our findings could be attributed to the small sample size and should be interpreted with caution.

There are several limitations to this analysis. This study analyzed an established cohort retrospectively who were managed by five different movement disorder specialists working in a group practice (PL, CS, BB, NP) and two neurosurgeons who implanted DBS (JS, EA). Management of stimulation parameters and adjuvant medications are not standardized between practitioners. Post-operative imaging to confirm lead location was not routinely performed though in the experience of the programming neurologist(s) the effect and side effect profiles suggested appropriate location. Additionally, standardized evaluations of CD were not completed routinely in follow-up which limited our ability to report motor outcomes in our cohort. Given our relatively small sample size, some of the comparisons that were performed were underpowered to demonstrate a difference. Larger prospective studies assessing the role of adjuvant therapies in the care of CD with DBS is recommended.

In this study we report the long-term outcomes of a relatively large cohort of CD patients treated with DBS and the role of adjuvant therapies to optimize symptom control. We recommend considering continuation of adjuvant therapies such as BoNT and medications for those patients whose symptoms are not optimally controlled with DBS monotherapy, especially during the early post-operative period when patient stimulation is not optimized.

## Data availability statement

The raw data supporting the conclusions of this article will be made available by the authors, without undue reservation.

## Ethics statement

The studies involving human participants were reviewed and approved by Henry Ford Health System Internal Review Board (IRB). Written informed consent for participation was not required for this study in accordance with the national legislation and the institutional requirements.

## Author contributions

AM-N and NP: conception and design of the study, acquisition of data, analysis, and interpretation of data. AM-N, CS, JW, and NP: drafting the article and revising it critically for important intellectual content. AM-N, CS, JW, JS, EA, PL, BB, PK, and NP: final approval of the version to be submitted. All authors have approved the final version of this article.

## Conflict of interest

Author CS has served on the Scientific Advisory Board of Abbvie, Acorda and Boston Scientific, has served as a consultant for Medtronic and Global Kinetics, and received research grants from Abbvie, Biohaven, and Michigan State University. Author JS receives honoraria from BCBSM for his role as a co-Director of MSSIC, research funding from Medtronic, SetPoint and Neuros, and compensation from NPA for his role on the Steering Committee for RAD-PD. Author EA is a consultant for Stryker, Inc and Functional Neuromodulation, Ltd. Author PL has served as a consultant or investigator in clinical trials sponsored by Acorda Therapeutics, Amneal, Appello, Axovant, Aptynix, Biogen, Biotie, Bukwang Pharmaceuticals, Cavion, Cerevel, Denali Therapeutics, F. Hoffmann LaRoche, Impax Laboratories Inc., Impel Neuropharma, Ipsen, Kyowa Kirin Hakko, Lundbeck A/S, The Michael J. Fox Foundation for Parkinson's Research, Neurocrine Biosciences, Pharma 2B, Mitsubishi Tanabe Neuropharma, Neurocrine Biosciences, NeuroDerm Ltd, Noven, Parkinson Study Group, Prexton Therapeutics, Revance, US WorldMeds, Saccadous, Sun Pharma, and Supernus, Revance Therapeutics, Saccadous, Supernus, and US WorldMeds. He has received speaker honoraria from Acorda Therapeutics, Britannia, the American Academy of Neurology, The International Parkinson's Disease and Movement Disorders Society, Kyowa Kirin Hakko, Neurocrine Biosciences, Pallidan Labs, US WorldMeds, and the World Parkinson Congress. He is compensated for services as editor-in-chief of Clinical Neuropharmacology and serves without compensation on the editorial boards of Journal of Neural Transmission, Translational Neurodegeneration, and Journal of Parkinson's Disease. Author BB has received honoraria as a consultant to Lundbeck and as a speaker for Neurocrine Biosciences. Author NP has received honoraria as a consultant to Acorda Pharmaceuticals, Revance Pharmaceutical and Abbott Laboratories, and as a speaker for Teva Pharmaceuticals. The remaining authors declare that the research was conducted in the absence of any commercial or financial relationships that could be construed as a potential conflict of interest.

## Publisher's note

All claims expressed in this article are solely those of the authors and do not necessarily represent those of their affiliated organizations, or those of the publisher, the editors and the reviewers. Any product that may be evaluated in this article, or claim that may be made by its manufacturer, is not guaranteed or endorsed by the publisher.
